# Plant Functional Genomics in A Few Days: Laser-Assisted Delivery of Double-Stranded RNA to Higher Plants

**DOI:** 10.3390/plants10010093

**Published:** 2021-01-05

**Authors:** Nabil Killiny, Pedro Gonzalez-Blanco, Siddarame Gowda, Xavier Martini, Ed Etxeberria

**Affiliations:** 1 Department of Plant Pathology, Citrus Research and Education Center, IFAS, University of Florida, Lake Alfred, FL 33850, USA; gowda@ufl.edu; 2 Department of Horticultural Sciences, Citrus Research and Education Center, IFAS, University of Florida, Lake Alfred, FL 33850, USA; pcgo@ufl.edu (P.G.-B.); eetxeber@ufl.edu (E.E.); 3 Department of Entomology and Nematology, North Florida Research and Education Center, Quincy, FL 32351, USA; xmartini@ufl.edu

**Keywords:** citrus, laser light, RNA interference, phytoene desaturase, functional genomics

## Abstract

The technology of transgenic plants is challenging and time consuming, especially for higher plants and trees such as citrus. Double-stranded RNA (dsRNA) delivery via a plant virus is an alternative method to create transgenic plants by suppressing the expression of plant endogenous genes. *Citrus tristeza* virus-based vector has been constructed specifically for use in citrus trees. However, this is time-consuming, as it can take up to nine months to produce the desired phenotype. Here we describe a much faster method for the study of gene function in citrus trees. In the current study, we used laser light for the delivery of dsRNA to citrus leaves. We targeted the endogenous reporter gene phytoene desaturase (*PDS*) and obtained the classical phenotype (leaf bleaching) in only three days after the laser-assisted delivery. Interestingly, the phenotype response was systemic, which indicates the movement of dsRNA and/or ssRNA within the plants. In addition, dsRNAs were taken up by phloem cells and the bleaching phenotype was clear around the main veins. In conclusion, the delivery of dsRNA to plants through laser treatment may provide a fast and more specific tool to study the gene function in higher plants and trees.

## 1. Introduction

Creating transgenic plants for plant improvement and resistance to diseases is slow and expensive. The traditional method of making transgenic plants requires many steps: defining and locating the desired genetic traits, isolating and multiplying the DNA fragments for insertion into the recipient plants, and growing and evaluating several generations of the modified plants to determine if the new genes have been passed on and are expressing the targeted traits. In higher plants such as trees, this can take several years to complete. RNA interference (RNAi) provides an alternative approach to obtain rapid changes in plant phenotype. RNAi uses exogenous double-stranded RNA (dsRNA) to stop or reduce production of a targeted protein by knocking down the complementary endogenous messenger RNA (mRNA) before translation. The enzyme Dicer cleaves the dsRNA into short double-stranded RNA fragments called small interfering RNA (siRNAs) that guide a large protein complex that silence mRNA [1,2].

Exogenous application of dsRNA would be the preferred method for agricultural application; however, this method is less efficient than direct injection. For instance, it takes two weeks for systemically detecting dsRNA in tomato leaves after exogenous application of dsRNA molecules [3]. Furthermore, this could take more time or be impossible if the host plant has a thicker cuticle. Therefore, there is a critical need to find a way to accelerate the uptake of exogenous dsRNA by plants.

It is particularly true in the case of citrus, where the thick cuticle makes it impossible for efficient exogenous application of dsRNA. The Florida citrus industry has been severely impacted by citrus greening disease, or Huanglongbing (HLB), a disease due to the phloem-limited ‘*Candidatus* Liberibacter asiaticus’ (*C*Las). As of today, all citrus varieties are susceptible to HLB, and disease-tolerant trees are few. HLB is spread by the Asian citrus psyllid, *Diaphorina citri*, the vector insect of *C*Las [4,5]. The bacterium multiplies in host trees and causes symptoms of blotchy mottle in the leaves, poor fruit quality, a high rate of fruit drop resulting in reduced yield, and eventual tree death [6]. We are now in the fifteenth year since HLB was first described [7], and nearly all of Florida’s citrus trees have been infected [8]. Therefore, every technology available to fight the disease and provide the citrus industry with more tolerant trees, including transgenic, is being utilized.

Virus-based vectors have been employed to speed the delivery of genetic material into the current generation of trees [9]. Virus-induced gene silencing (VIGS) takes advantage of the host plant’s own natural defense mechanisms to suppress intruding viruses, which are themselves usually single strands of RNA. Since 1998, viruses such as tobacco mosaic virus [10], potato virus X [11], and tobacco rattle virus [12] have been used with VIGS for delivery of endogenous plant genes.

Recently, mild strains of citrus tristeza virus (CTV), a specific virus of citrus, have been selected to produce dsRNA [13]. For example, CTV-based vector was used to deliver a truncated abnormal wing disc construct (t*AWD*) to citrus plants. While the small RNA was successfully transferred to plant phloem, it was only 15% effective in causing abnormal wings in adult *D. citri* [9]. The construct can also be delivered by topical application directly to insects or by feeding [14], but this is not practical for field use.

Coupling target constructs with the truncated gene for phytoene desaturase (*PDS*) or tagging with green fluorescent protein (GFP) has been popular with VIGS. Suppressing the PDS enzyme inhibits carotenoids biosynthesis and increases chlorophyll degradation, resulting in distinctive yellow streaks and spots (photobleaching), which can be used as a marker, confirming that the constructs were delivered to the host plant. Graft-inoculation of CTV-t*PDS* takes many weeks for the phenotype to begin appearing in new shoots. While faster than traditional transgenics, VIGS still takes weeks to months to achieve.

To increase penetration of dsRNA, and accelerate the gene silencing, we examined the use of laser light (light amplification by simulated emissions radiation) to puncture microscopic holes within the lipidized leaf surface without extensive damage to the leaf tissue. Since 2005, laser micro-perforation has been used to introduce exogenous DNA [15] and small molecules such as glucose, lysine, trehalose, and nanoparticles [16] into plant cells. In the current work, we introduced laser light micro-perforation as a fast and reliable alternative method of introducing dsRNA into citrus leaves. For proof of concept, *PDS* gene was again targeted for silencing due to the visible phenotype.

## 2. Results and Discussion

### 2.1. Laser Generated Perforations

**Figure 1:**
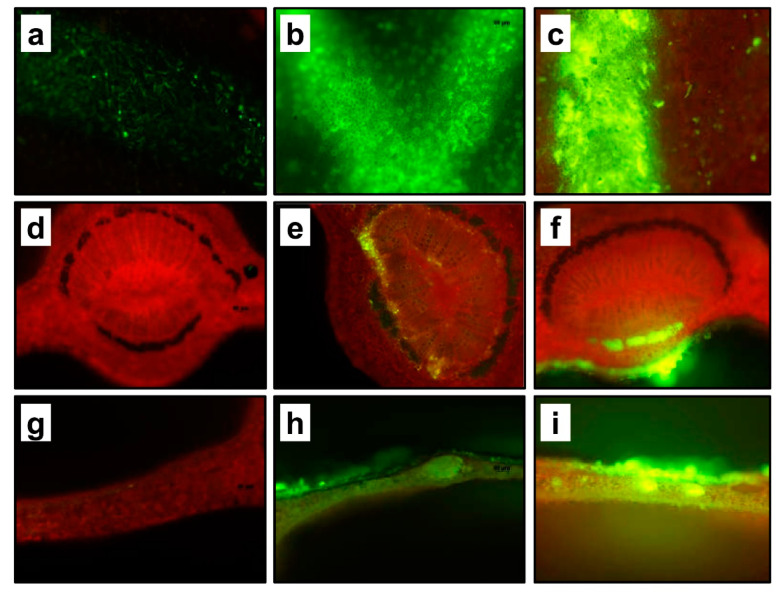
**Delivery of dsRNA to citrus phloem through laser micro-perforation.** (**a**–**c**) Fluorescence micrographs of laser-perforated areas on citrus leaves and applied with H_2_O, deoxyglucose, 2-NBDG, and CY-3 labeled dsRNA-*PDS*, respectively. (**d**–**f**) cross sections of laser-treated citrus leaf petioles and applied with H_2_O, deoxyglucose, 2-NBDG, and CY-3 labeled dsRNA-*PDS*, respectively. (**g**–**i**) cross sections of lased-treated citrus blades and applied with H_2_O, deoxyglucose, 2-NBDG, and CY-3 labeled dsRNA-*PDS*, respectively. Note the localization of fluorescent deoxyglucose, 2-NBDG, and the CY-3 labeled dsRNA molecules are localized in the phloem (**e**,**f**). Control leaves were laser-treated, then water was applied instead of dsRNA or fluorescent glucose.

Laser light created small pores on the leaf cuticle (Figure 1a). Each perforation across the cuticle had an average surface area of 3.14 × 10^−4^ cm^2^. The laser pattern of 150 perforations per rectangle represents an exposed total area of 0.047 cm^2^ per rectangle or 0.470 cm^2^ per leaf.

### 2.2. Leaf Penetration and Mobility of Experimental Solutions

No fluorescence was detected when H_2_O was applied on laser perforation (Figure 1a,d,g). Our initial testing was to apply on laser perforation rectangles deoxyglucose, 2-[ N -(7-nitrobenz2-oxa-1,3-diazol-4-yl)amino]-2-deoxyglucose (2-NBDG), a fluorescent glucose analog already demonstrated to be taken up by plant cells [16,17]. Glucose rapidly penetrates the leaf tissue through laser-generated perforations and subsequently migrates to the phloem. A cross section of the petiole three days after treatment showed a strong fluorescent signal within the vascular tissue, specifically in the phloem (Figure 1b,e,h). A previous study demonstrated that glucose entered laser perforation pores, and moved to the apoplastic spaces before being taken up by phloem cells [16]. In agreement with the previous results, we also found that most of the applied dsRNA migrated to the phloem, as demonstrated by the petiole cross section of citrus leaves following laser perforation and exogenous application of dsRNA (Figure 1c,f,i).

### 2.3. Phenotype Changes and Gene Expression

**Figure 2:**
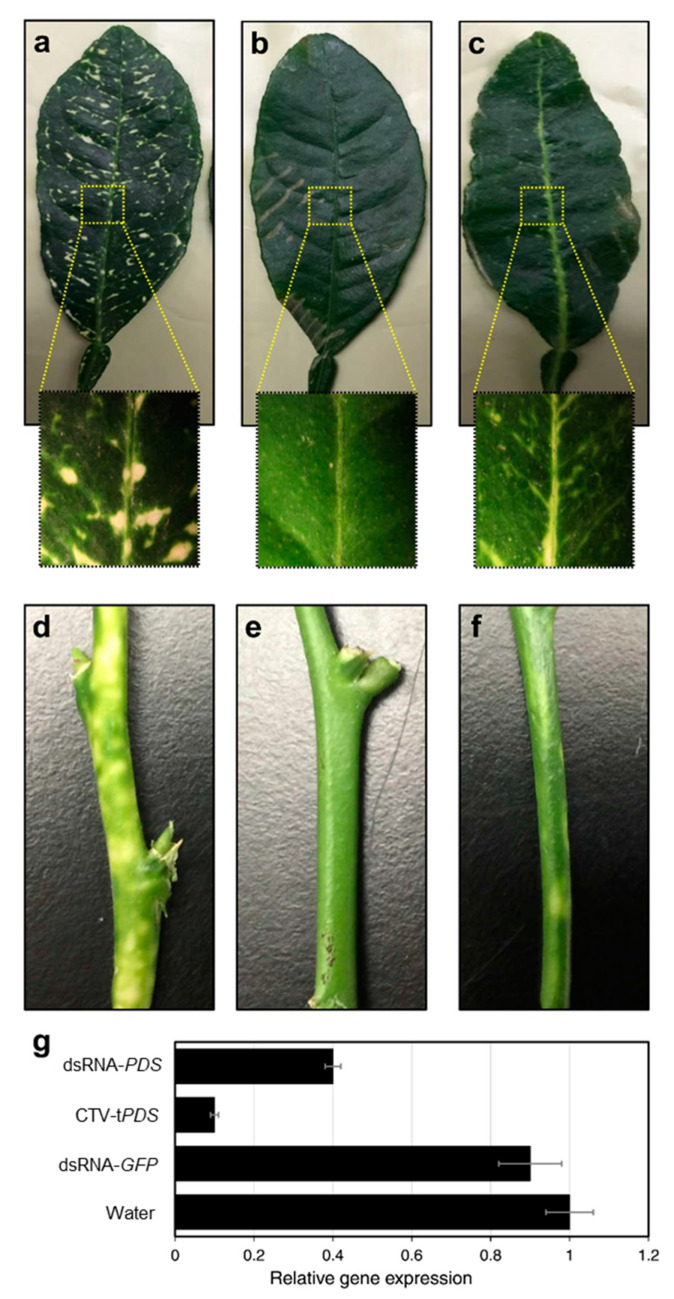
Color bleaching and relative gene expression of *PDS* in citrus leaves after delivery of dsRNA-*PDS* thought laser micro-perforation. (**a**–**c**) Photobleaching in leaves of CTV-t*PDS* inoculated, water control, and dsRNA-*PDS* laser-delivered plants, respectively. Leaves were harvested from CTV-t*PDS* plants after six months of inoculation, whereas they were collected from laser-treated plants after three days of laser treatment. (**d**–**f**) Photobleaching in stems of CTV-t*PDS* inoculated six months after treatment, water control, and dsRNA-*PDS* laser-delivered plants three days after treatment, respectively. Note that bleaching in dsRNA-*PDS* laser-delivered only occurred in the veins. (B and E) show the dsRNA-*GFP* treatment does not induce the photobleaching phenotype, like non-treated control (not shown). (**g**) Relative gene expression of *PDS* in citrus trees three days after laser treatment and dsRNA application. Control plants were applied with water after the laser treatment. Bars represent standard errors. *N* = 15.

We observed color bleaching, the characteristic phenotype of *PDS* silencing in both dsRNA-*PDS* laser-delivered and CTV-t*PDS* inoculated plants (Figure 2a–c,d–f). Control plants and laser-delivered dsRNA-*GFP* plants did not express bleaching symptoms (Figure 2b,e), indicating that bleaching symptoms were only due to *PDS* silencing by dsRNA-*PDS*. Importantly, bleaching appeared only after three days on laser-delivered dsRNA-*PDS* plants, whereas it took up to nine months to have bleaching symptoms with CTV-t*PDS* inoculation. In CTV-t*PDS* inoculated plants, bleaching symptoms were scattered on the entire surface of the leaf according to the movement of the CTV vector, whereas bleaching symptoms were only apparent around leaf veins after laser delivery (Figure 2a–c). This confirms the results from the cross-section of the petiole showing fluorescence only in the phloem tissue. Gene expression analysis showed a significant difference among treatments (*p* < 0.05). Control plants and dsRNA-GFP inoculated plants had no decrease of *PDS* expression, whereas both laser-delivered dsRNA-*PDS* plants and CTV-t*PDS* inoculated plants had a significant reduction in *PDS* expression (Figure 2g). The reduction in *PDS* expression was higher for CTV-t*PDS* inoculated plants than for dsRNA-*PDS* laser-delivered plants. The reduction in the gene expression agreed with the observed phenotype. The CTV-t*PDS* inoculated plants showed higher bleaching symptoms than those treated with dsRNA-*PDS* after laser treatment. The low level of bleaching symptoms observed in dsRNA-treated plants after laser perforation could be attributed to the limited amount of applied dsRNA (10 µg pr leaf). On the other hand, more dsRNAs are generated in the CTV-t*PDS* inoculated plants, because the viral RNA polymerases produce more dsRNAs each time the virus vector replicates [9].

Delivery of exogenous dsRNA into the plant cell or plant vascular system is a challenging procedure. Several methods have been used to deliver exogenous dsRNA into plant vascular system including mechanical inoculation, spraying, root or seed soaking, trunk injections, and infiltration [18]. Unfortunately, low- and high-pressure spraying were shown to be ineffective [19]. Previous studies suggested that dsRNAs either were destroyed or retained on the leaf surface or in the mesophyll apoplast upon spraying [19]. Several techniques were also developed to enhance the delivery of dsRNA into plants including the use of clay nanosheets, cationic nanoparticles, peptides, and surfactants [18]. A previous study also showed that the stability and delivery of dsRNA was significantly improved after being loaded into layered double hydroxide nanoparticles (BioClay) [20]. From the experiments conducted, we observed that micro-perforation of the plant cuticle with a laser light accelerated the uptake and the expression of dsRNA. According to our knowledge, this is the first time that laser-induced micro-perforation has been used to facilitate the acquisition of dsRNA. The classical method used to modify the phenotype of a citrus plant with the use of dsRNA can take up to nine months. In the current study, the appearance of photobleaching in the laser-treated seedlings occurred within three days. Therefore, we demonstrated that the micro-perforation of the leaf cuticle with a laser beam prior to dsRNA exposure dramatically reduces the time until the plants show symptoms of silenced genes. In addition, expression of the altered phenotype was systemic across the plant and not localized only to the proximity of the laser micro-perforations. It is likely that this method could be applied to a wide range of plants to accelerate the uptake of dsRNA. Compared to CTV-virus based delivery of the same t*PDS*, this achievement represents a giant leap.

## 3. Material and Methods

### 3.1. Plant Material

Alemow (*Citrus macrophylla*) used as citrus plant material in experiments were about 60 ± 5 cm tall, around 12 months old, and maintained in an approved USDA-APHIS/CDC-secured greenhouse, at 22–24 °C; 60 ± 5% RH; 16:8 L/D photoperiod, at the Citrus Research and Education Center (CREC), University of Florida, Lake Alfred, Florida. Plants were irrigated twice weekly and fertilized once weekly, using 20-10-20 NPK fertilizer (Allentown, PA, USA). As controls, we used alemow plants with photobleaching phenotype, which were produced in an earlier study using the *Citrus tristeza virus*-based silencing vector clone including the truncated phytoene desaturase gene (CTV-t*PDS*) [9].

### 3.2. Synthesizing of the dsRNA

*PDS* (phytoene desaturase) gene was amplified from total RNA extracted from *Citrus macrophylla* using SuperScript^®^ III One-Step RT-PCR System with Platinum^®^ Taq DNA Polymerase (Life Technologies, Carlsbad, CA, USA). Primers were designed based on *C. sinensis PDS* gene (Genbank accession no. DQ235261.1). *PDS*-PacI (5′-CGAGTTAATTAAAGCCTTTGCTTCAGCGTTTCTGAAAGTGCTTTC-3′) and *PDS*-StuI (5′-GACAAGGCCTGTCTCATACCAGTTCCCGTCCCCATCTTTCC-3′) were designed to amplify a truncated fragment corresponding to the nucleotides 4–395 of the *PDS* gene. The amplified fragment (391 nts) was gel purified using GENECLEAN III kit (MP Biomedicals) and used for TA cloning into pGEM-T vector (Promega, Madison, WI, USA). *PDS*/pGEM-T vector was sequenced using universal T7 promoter primer to confirm the cloned *PDS* sequence. *PDS*/pGEM-T vector was linearized by restriction digesting with NcoI or SpeI to generate sense and antisense transcripts using Ambion^®^ MAXIscript^®^ T7/SP6 In Vitro Transcription Kit (Life Technologies, Carlsbad, CA, USA), respectively. To produce *PDS*-dsRNA, sense and antisense transcript were annealed in a single tube by denaturing at 70 °C for 10 min followed by slow cooling to room temperature for 20 min. To eliminate the DNA template and single-stranded RNAs, annealed dsRNA was treated with RQ DNase I (Promega, Madison, WI, USA) and RNase A (Sigma-Aldrich, St. Louis, MO, USA). The resulting dsRNA was extracted with phenol:chloroform:isoamyl alcohol (25:24:1, *v/v*). The amount of purified dsRNA was measured by the NanoDrop 2000 spectrophotometer (ThermoFisher Scientific, Waltham, MA, USA). Specific primers (Forward primer with SpeI: 59- GCGAACTAGTATGGC- TAGCAAAGGAGAAGAACTTTTCACTG -39 and Reverse primer with SacII: 59- GAGACCGCGGCTACCCCTCGAGT- TATTTGTAGAGCTCATC -39) were used to amplify full-length *GFP* gene by using TMV-30BGFP.

### 3.3. Labeling of the dsRNA

Coupling the fluorescent label, Cy3 to long dsRNA was performed using *Silencer*^®^ siRNA Labeling Kit with Cy™3 dye (Ambion, Austin, TX, USA) according to the protocol provided by the manufacture.

### 3.4. Laser Treatment

Leaves were laser-treated using a low-energy carbon dioxide laser etching machine (model XY Mark-10; GPD Technologies, Shenzhen, China) located at the University of Florida’s Citrus Research and Education Center in Lake Alfred, FL. Laser specifications used were those already reported for citrus fruits [21]. We used the dot matrix pattern [16] and the energy per surface area of one dot = 0.00785 W/dot/10^−6^ s. The laser-perforated area consisted of five successive rectangles (15 rows of 10 perforations each for a total of 150 perforations per rectangle). There were two laser-perforated areas per leaf for a total of 10 perforation rectangles per leaf (1500 perforations total).

Leaves were laser-treated once on each side of the mid-vein. Immediately after laser perforation, 10-20 µl of test solutions were manually applied to each perforated area. After the solution dried, a layer of mineral oil (light white oil, M-3516; Sigma, St. Louis, MO, USA) was applied to prevent desiccation. All leaves were cleaned before observation in the microscope to eliminate background fluorescence produced by any unabsorbed solutions.

### 3.5. Experimental Solutions

Our initial testing was carried out with 2-[N-(7-nitrobenz-2-oxa-1,3-diaxol-4-yl)amino]-2-deoxyglucose (2-NBDG), a fluorescent glucose analog already demonstrated to be taken up by plant cells [17], at concentration of 5 mg mL^−1^. For dsRNA solutions, we used 500 ng µL^−1^, 20 µL per leaf and three leaves at the base of each plant were treated.

### 3.6. Microscopy

Microscopic observations were made using a Carl Zeiss Axion Scope A-1 equipped with a Canon EOS Rebel T3i camera and a Carl Zeiss AxioCam ICc1. Low magnification images were taken with a Zeiss Stemi SV11 fluorescent stereoscope (Carl Zeiss Microscopy, Oberkochen, Germany).

### 3.7. RNA Extraction and Gene Expression

TriZol^®^ reagent (Ambion^®^, Austin, TX, USA, Life Technologies, Carlsbad, CA, USA) was used to extract total RNA from samples. The quantity and quality of isolated RNA were assessed using a NanoDrop 2000 spectrophotometer (Thermo Scientific, Waltham, MA, USA). RNA was extracted three days after treatment. SuperScript first-strand synthesis system (Invitrogen, Carlsbad, CA, USA) with random hexamer primers as described by the manufacturer’s instructions was used to synthesize cDNA. SYBR Green PCR master mix (Applied Biosystems, Foster City, CA, USA) was used to perform the qPCR on an ABI 7500 Fast-Time PCR System (Applied Biosystems, Foster City, CA, USA). Samples were analyzed in triplicate for each biological replicate for each treatment. Five biological replicates (trees) were used. The primers *PDS*-RT-F (5′-GGACGGGAACTGGTATGAGA-3′) and *PDS*-RT-R (5′-TGGCCAATATCCCATTTAGC-3′) were used to measure the gene expression. The relative expression of *PDS* was measured according to the 2^−ΔΔ*C*T^ method [22]. Normalization of gene expression was performed using GAPDH as a reference gene. Differences in gene expression between treatments were analyzed with ANOVA after ensuring normal distribution of the data and equal variance between treatments. Separation of means was conducted with a Tukey test.

## 4. Conclusions

Efficient delivery of double-stranded RNA against targeted genes has been a challenge, especially in higher plants. Using laser-assisted delivery of double-stranded RNA to citrus, we depressed the expression of the phytoene desaturase gene, resulting in a leaf-bleaching phenotype in only a few days instead of up to a year or more, when transgenic or virus-induced gene silencing approaches are used. In the era where Huanglongbing disease is devastating the worldwide citrus industry, time is critical, and rapid approaches to functional genomics will greatly accelerate our understanding of the disease and assist in finding sustainable solutions. Finally, the innovative method described could be employed to deliver dsRNA targeting disease-carrying hemipteran insects, as well as being a powerful tool for functional genomics studies in higher plants.

## Data Availability

All data are contained within the article.

## Abbreviations

*C*Las	*Candidatus* Liberibacter asiaticus
*dsRNA*	Double stranded RNA
GFP	Green fluorescent protein
HLB	Huanglongbing
mRNA	Messenger RNA
2-NBDG	2-[ N -(7-nitrobenz2-oxa-1,3-diazol-4-yl)amino]-2-deoxyglucose
*PDS*	*Phytoene desaturase*
RNAi	RNA interference
siRNAs	Small interfering RNAs
VIGS	Virus-induced gene silencing
